# Unbalanced chromosome 1 abnormalities leading to partial trisomy 1q in four infants with Down syndrome and acute megakaryocytic leukemia

**DOI:** 10.1186/1755-8166-2-7

**Published:** 2009-02-19

**Authors:** Maria Luiza Macedo Silva, Maria do Socorro Pombo-de-Oliveira, Susana C Raimondi, Hasmik Mkrtchyan, Eliana Abdelhay, Amanda Faria de Figueiredo, Mariana Tavares de Souza, Daniela Ribeiro Ney Garcia, Eliane Maria Soares de Ventura, Adriana Martins de Sousa, Thomas Liehr

**Affiliations:** 1 Department of Cytogenetic, The National Center for Bone Marrow Transplantation (CEMO-INCa), National Cancer Institute (INCa), Rio de Janeiro, RJ, Brazil; 2 Department of Experimental Medicine, National Cancer Institute (INCa), Rio de Janeiro, Brazil; 3 Department of Pathology, St Jude Children's Research Hospital, Memphis, TN, USA; 4 Institute of Human Genetics and Anthropology, Jena, Germany; 5 Pediatric Oncohematology Center, Hospital Oswaldo Cruz, Pernambuco University, Recife, Brazil; 6 Martagão Gesteira Institute of Pediatrics and Child Development, Federal University of Rio de Janeiro, Rio de Janeiro, RJ, Brazil

## Abstract

**Background:**

Children with Down syndrome (DS) have an increased risk of childhood acute leukemia, especially acute megakaryoblastic leukemia (AMKL) also called acute myeloid leukemia (AML) type M7. Here four yet unreported infants with such malignancies are reported.

**Results:**

An unbalanced translocation involving chromosome 1 was identified by GTG banding in all cases. These were characterized in more detail by molecular cytogenetic approaches. Additional molecular analysis revealed in three of the four cases mutations in exon 2 of the GATA binding protein 1 (globin transcription factor 1), located in Xp11.23.

**Conclusion:**

Our results corroborate that abnormalities of chromosome 1 are common in DS-associated AMKL. Whether this chromosomal region contains gene(s) involved in hematopoietic malignant transformation remains to be determined.

## Background

Among congenital disorders, Down Syndrome (DS) is one of the most common, affecting 1/800 – 1/1000 live births. Children with DS have an increased risk of childhood acute leukemia (AL) when compared to the general pediatric population under 4 years of age [1]. In DS the majority of leukemia diagnosed below the age of 2 years is acute megakaryoblastic leukemia (AMKL) or acute myeloid leukemia (AML) type M7, according to the French-American-British classification. This neonatal leukemia is usually indistinguishable from other AL in clinical, cytological and immunophenotypical aspects. Nearly 25% of infants that undergo remission after a transient leukemia episode develop an AL 1–3 years later. Approximately 20% of infants with this malignancy progress to a sub-type of AML-M7 or AMKL [2].

Several reports have now suggested that mutations in the hematopoietic zinc-finger transcription factor gene GATA1, which is essential for proper development of erythroid cells, megakaryocytes, eosinophilis and mast cells, could be an initiating event in DS leukemogenesis [3,4]. Besides the involvement of GATA1, trisomy 21 is strongly associated with leukemogenesis. Cytogenetic analyses revealed other acquired recurrent abnormalities associated with gain of chromosome 21. Recently, Forestier and co-workers [5] analyzed 189 DS-associated AML cases (DS-AML) and confirmed a distinct entity, originating from other genetic pathways than non-DS-AML.

Overall, children with DS are uniquely predisposed to clonal disorders affecting the megakaryocyte lineage. At birth, they can present hematopoiesis characterized by pancytopenia and a myelodysplastic syndrome (MDS) can be diagnosed. Some authors describe that this condition develops to TMD (= transient myeloid disorder) also called transient leukemia (TL) [6]. Even though the presence of trisomy 21 and GATA1 mutations appear to be sufficient for the excessive proliferation of megakaryoblasts seen in TMD, it seems to be insufficient for leukemogenesis because the majority of TMD resolve spontaneously. The spontaneous disappearance of these immature cells shortly after birth suggests that a specific environment might be essential for the proliferation of these cells. A hypothesis to explain this is that this excessive proliferation of megakaryoblasts arises from the fetal liver. The spontaneous regression of TMD shortly after birth could then be explained by the loss of a permissive fetal hematopoietic environment [6]. TMD and AMKL are associated with trisomy 21 and mutations in GATA1. However, it has been speculated that other additional lesions result in explicitly leukemia. These additional lesions could be mutations in P53, altered telomerase's activity, or additional acquired karyotype abnormalities, trisomy 8 being the most common in DS-AMKL [7-10].

Here four children with as DS-AML were studied for presence of mutations in GATA1, GTG banding and molecular cytogenetic studies. Besides that 3/4 cases had a GATA1 mutation also 3 of them were associated with an unbalanced rearrangements of chromosome 1.

## Case presentation

Between 2005 and 2006, four DS children with history of MDS were referred to the cytogenetic department at Instituto Nacional de Câncer (INCa) of Rio de Janeiro, Brazil. The clinical data, including outcome, as well as molecular and (molecular) cytogenetic results are summarized in Tab. 1.

**Table 1:** Clinical details and results obtained in the four studied cases

	**Case 1**	**Case 2**	**Case 3**	**Case 4**
**Clinical History**	persistent anemia since birth	anemia, thrombocytopenia, MDS	anemia, thrombocytopenia, MDS	anemia, thrombocytopenia, MDS
**Age of diagnosis**	20 months	20 months	17 months	11 months
**Sex/Ethnicity**	male/non-white	female/white	female/non-white	male/non-white
**Diagnosis**	August 2005	March 2006	February 2006	May 2005
**BM Morphology**	Hyper-cellular, 72% blast cells, abnormal nucleoli and blebs	Hyper-cellular, 95% blast cells	Hyper-cellular, 60% blast cells	Hyper-cellular, 80% blast cells, nucleoli and blebs
**WBC [ml]**	3.8 × 10^3^	13.0 × 10^6^	4.0 × 10^6^	36.7 × 10^6^
**Blast cells [%]**	45	24	10	43
**Platelets count [ml]**	90 × 10^6^	26.0 × 10^6^	46 × 10^6^	90 × 10^6^
**Final Diagnosis**	AML-M7	AML-M7	AML-M7	AML-M7
**Immunophenotype**	CD33/CD13+; CD7/CD34/CD117+; CD61/CD41+	CD61/CD14+/CD13/CD33+; CD38/CD117+	CD33/CD13+; CD7/CD34+; CD61+	CD33/CD13+; CD7/CD34/CD117+; CD61/CD41+
**Cytogenetics G banding**	47,XY,der(19)t(1;19) (q24;p13.3),der(20)t(1;20) (q24;q11.2),+21c [13]	48,XX,t(1;16)(q21;q12.1), +der(16)t(9;16)(q13;q12.1), +21c [15]	47,XX,der(17)t(1;17) (q32;p13),+21c [20]	47,XY,-1, add(5)(p14), del(7)(p15), +der(7)t(1;7)(q21;p22), +21c [10]
**MCB Banding**	47,XY,der(19)t(1;19) (q31;p13.3),der(20)t(1;20) (q31;q12~13.1),r(20) (p11.2q12),+21c [53]/47,XY, der(2)t(2;11)(q37.3;q12~13), der(19)t(1;19)(q31;p13.3), der(20)t(1;20)(q31;q12~13.1),+21c[5]	48,XX,t(1;16)(q31~32;q23), der(5)t(1;5)(q31~32;p13),+der(16)t(16;1;5) (q23;q31~32;p13),+21c	47,XX,der(17)t(1;17) (q32;p13),+ 21c	n.a.
**Gata-1 *status***	no mutation	c.56G > A	c.113 del G	c.201A > G
**Treatment**	AML BFM 98	AML BFM 98	AML BFM 98	AML BFM 98
**Disease status**	Dead with disease	Alive in complete remission	Alive in complete remission	Dead in complete remission

Clinical details and results obtained in the four studied AML-M7 with Down syndrome cases are given. Abbreviations: AML = acute myeloid leukemia; AML-M7: acute megakaryoblastic leukemia; BFM: Berlin-Frankfurt-München; BM = bone marrow; n.a. = not available due to lack of material; MDS = myelodysplastic syndrome; WBC: white blood cell count; MCB: multicolor banding

The infants were 11 to 20 months old. In all four cases the initial diagnosis was established by cell morphology, cytochemistry and immunophenotyping analysis as standardized procedures [11]. Criteria for AMKL diagnosis was the presence of megakaryocyte-specific membrane markers (CD41, CD42a, CD61) or CD36 positive bone marrow (BM) fibrosis (Tab. 1). The diagnosis of MDS was established previously in cases 2–4 according to the WHO criteria, where one of the main distinguishing features of these conditions is the proportion of blast cells in the peripheral blood (PB) and/or BM as well as specific cytogenetic alterations [12]. Case 1 was admitted with AMKL but had been treated for five months with iron orally, because of refractory anemia. The status of bone marrow of the 4 cases was hyper-cellular, with high percentage of CD13, CD33, CD61 and CD41 positive blast cells. As the diagnosis was AMKL/AML subtype M7 according to FAB classification [13] all four patients were treated according to the AML BFM 98 protocol.

At present (September 2008), only 2 patients are alive and in complete remission (cases 2 and 3). Patient with #1 died due to a relapse and #4 was in remission, but died of bronchial aspiration.

## Results and discussion

It is well known that DS-children are predisposed to clonal disorders affecting the megakaryocyte lineage [6]. Also TMD and AMKL are associated with trisomy 21 and GATA1 mutations. Table 1 summarizes each of our four patient's data concerning clinic, (molecular) cytogenetics and response to treatment.

GATA 1 mutation in exon 2 could not be detected for case 1, but were present in the other 3 cases (#2, #3, #4). These findings are important to show the involvement of this gene in our cases with DS-AMKL [3].

Chromosomal breakpoints detected in banding cytogenetics were confirmed and refined by molecular cytogenetics. Interestingly, three of the four cases (cases 1–3), break-events took place in the chromosomal region 1q31 to 1q32. In case 4 also chromosome 1q was involved in a rearrangement – here the breakpoint could not be refined by molecular cytogenetics due to lack of material. Recently, it has been shown that chromosomal aberrations could provide important clues to the genetic events associated with the transformation of a pre-leukemic, possibly GATA1 positive clone. The aberrations found for the 1q31~32 region, especially duplication, are in concordance with previous reports of DS-AML with GATA1 mutations [5]. Also partial duplications of 1q are reported as typical, which we can confirm here for 3/4 cases. Additionally detected unbalanced rearrangements involved chromosomes 2, 5, 7, 11, 16, 17 and 19, which were also in concordance with previous reports on DS-AML.

Overall, our results, support evidence that genes located at region 1q31 and 1q32 are responsible for secondary or even primary mechanisms for the origin of AMKL in DS. Further gene-hunting studies in this region have to be performed to elucidate the pathogenetic mechanisms of the long arm of chromosome 1 in DS-AML.

## Methods

### Cytogenetics

#### Banding cytogenetics

**Figure 1:**
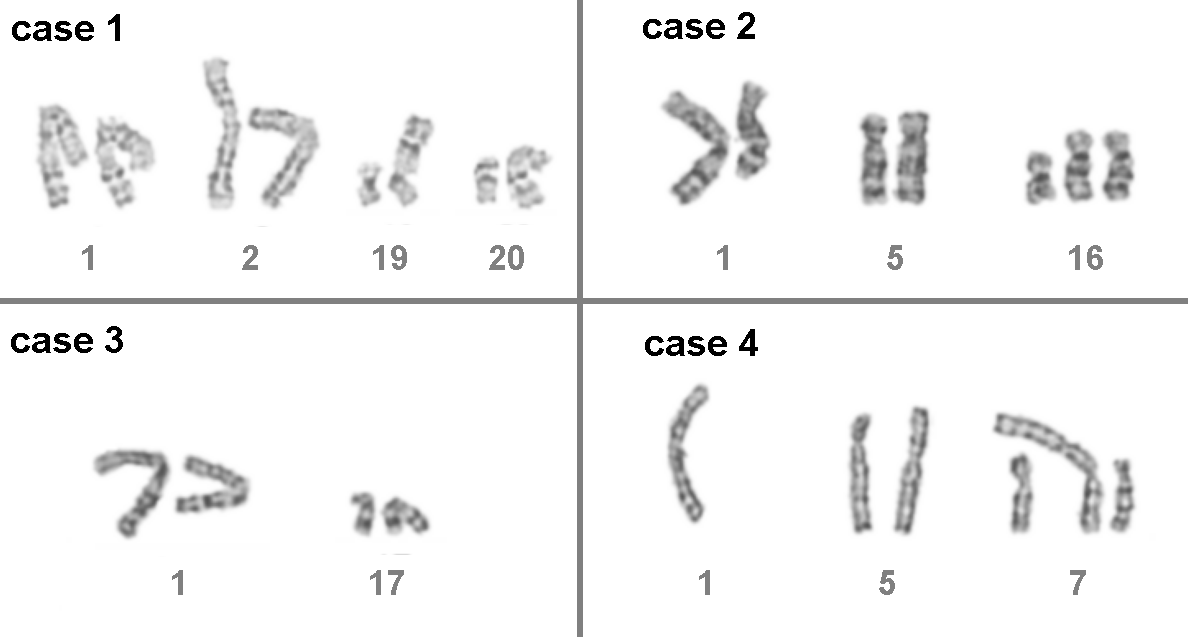
**Partial karyotypes presenting the aberrant cytogenetic banding results obtained in the four studied cases; the additional constitutional chromosomes 21 are not depicted**. case 1: 47,XY,der(19)t(1;19)(q24;p13.3),der(20)t(1;20)(q24;q11.2),+21c. case 2: 48,XX,t(1;16)(q21;q12.1),+der(16)t(9;16)(q13;q12.1),+21c. case 3: 47,XX,der(17)t(1;17)(q32;p13),+21c. case 4: 47,XY,-1,add(5)(p14),del(7)(p15),+der(7)t(1;7)(q21;p22),+21c.

Karyotypes of BM cell were obtained at the time of AMKL diagnosis for all four cases [See Figure 1]. Cytogenetic analysis was performed as described [14]. Chromosomes were identified and analyzed in concordance with [15].

#### Molecular cytogenetic analysis

**Figure 2:**
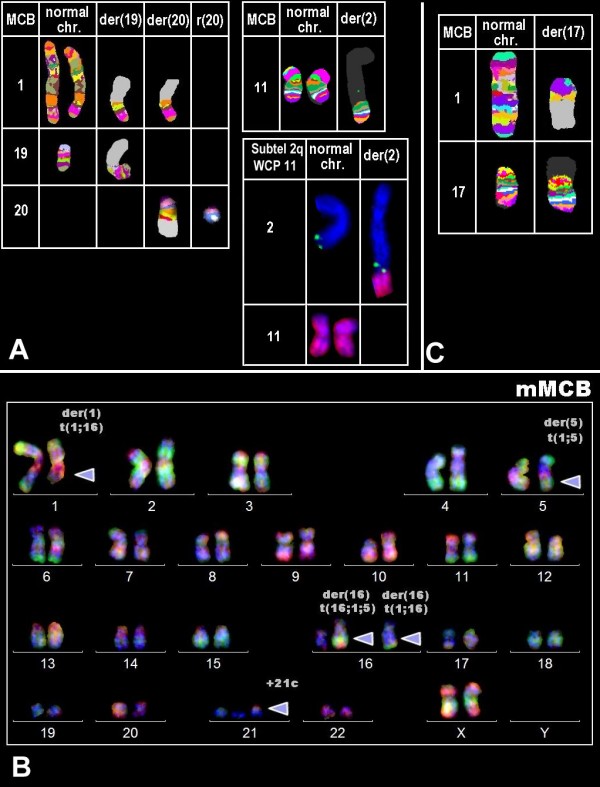
**FISH-results obtained in cases A, B and C after application of mMCB and MCB**. case A: MCB probe sets 1, 19 and 20 showed the presence of two normal chromosomes 1, one normal chromosome 19 and no normal chromosome 20. Additionally a der(19)t(1;19)(q21;p13.3), a der(20)t(1;20)(q21;q11.2) and an r(20)(p11.2q12). A der(2)t(2;11) was described using MCB 11 and subtelomeric probe 2q (latter result not shown). case B: mMCB identified a reciprocal translocation t(1;16)(q31;q23) (red arrowhead), a der(5)(1;5)(q32;p13) (blue arrowhead), a der(16)t(16;1;5)(q23;q31~q32;p13) (green arrowhead) and the constitutional additional chromosome 21 (grey arrowhead). mMCB result is shown here in a three color channel depiction. case C: MCB using probe sets for chromosomes 1 and 17 confirmed the presence of a unbalanced translocation t(1;17)(q32;p13).

To detect possible cryptic chromosomal changes multiplex fluorescence in situ hybridization (M-FISH) [16] and (multitude) multicolor chromosome banding (mMCB), MCB for chromosomes 1, 11, 17, 19 and 20 [17,18] were applied [see Figure 2]. Additionally the following bacterial artificial chromosome (BAC) probes were applied: RP11-172J6 in 1q22, RP11-75C23 in 1q31, RP11-415M14 in 1q25, RP11-75C23 in 1q31 and RP11-031A5 in 5p12. For-probe-labeling refer to [16,18]. Per case and probe between 5 and 25 metaphases were analyzed, each.

#### Molecular genetic analysis

dHPLC (denaturating high performance liquid chromatography) technique and direct sequencing were done to detect and characterized possible GATA1 mutations [19].

### Ethical approval

The Ethical Committee (Instituto Nacional de Câncer (INCa) of Rio de Janeiro, Brazil) approved this study (CONEP #12087).

## Competing interests

The authors declare that they have no competing interests.

## Authors' contributions

MdSPdO, EMSdV and AMdS provided the bone marrow samples of the 4 cases and their clinical history. MLMS, AFdF, MTdS, EA, DRNG and ScR, did the cytogenetic work up and analysis of the probes and the karyotype interpretation. HM and TL did the FISH and further MCB-analysis. All coauthors have been involved in drafting the manuscript. EA, TL, MLMS and HM revised it critically for important intellectual content.
